# Predictors of Broad Dimensions of Psychopathology among Patients with Panic Disorder after Cognitive-Behavioral Therapy

**DOI:** 10.1155/2018/5183834

**Published:** 2018-03-12

**Authors:** Sei Ogawa, Masaki Kondo, Keiko Ino, Risa Imai, Toshitaka Ii, Toshi A. Furukawa, Tatsuo Akechi

**Affiliations:** ^1^Department of Psychiatry and Cognitive-Behavioral Medicine, Nagoya City University Graduate School of Medical Sciences, Nagoya, Japan; ^2^Department of Health Promotion and Human Behavior, Kyoto University Graduate School of Medicine/School of Public Health, Kyoto, Japan

## Abstract

**Background:**

Many patients with panic disorder meet criteria for at least one other diagnosis, most commonly other anxiety or mood disorders. Cognitive-behavioral therapy is the best empirically supported psychotherapy for panic disorder. There is now evidence indicating that cognitive-behavioral therapy for panic disorder yields positive benefits upon comorbid disorders.

**Objectives:**

The present study aimed to examine the predictors of broad dimensions of psychopathology in panic disorder after cognitive-behavioral therapy.

**Methods:**

Two hundred patients affected by panic disorder were treated with manualized group cognitive-behavioral therapy. We examined if the baseline personality dimensions of NEO Five Factor Index predicted the subscales of Symptom Checklist-90 Revised at endpoint using multiple regression analysis based on the intention-to-treat principle.

**Results:**

Conscientiousness score of NEO Five Factor Index at baseline was a predictor of four Symptom Checklist-90 Revised subscales including obsessive-compulsive (*β* = −0.15, *P* < 0.01), depression (*β* = −0.13, *P* < 0.05), phobic anxiety (*β* = −0.15, *P* < 0.05), and Global Severity Index (*β* = −0.13, *P* < 0.05).

**Conclusion:**

Conscientiousness at baseline may predict several dimensions of psychopathology in patients with panic disorder after cognitive-behavioral therapy. For the purpose of improving a wide range of psychiatric symptoms with patients affected by panic disorder, it may be useful to pay more attention to this personal trait at baseline.

## 1. Background

Panic disorder is associated with substantial reductions in social functions and lifetime prevalence rates are approximately 3% [1]. Generally, panic disorder has broad dimensions of psychopathology. Fifty to eighty percent of patients affected by panic disorder meet criteria for other psychiatric diagnoses, mainly other anxiety or mood disorders [2, 3]. This overlap among the disorders may occur owing to general distress or negative affectivity, a shared genetic predisposition and a common neurobiology [4]. The comorbidity between panic disorder and depression is considered to be related to symptom severity and poorer treatment outcome [5]. Panic disorder with comorbid psychiatric symptoms is regarded as a severe condition. Moreover, panic disorder shows subthreshold psychiatric symptoms such as somatization, phobic anxiety, interpersonal anxiety, and depression [6]. Successful treatment of panic disorder is associated with improvement in broad dimensions of psychopathology [7].

The efficacy of cognitive-behavioral therapy has been established for panic disorder [8, 9]. A number of studies presented that cognitive-behavioral therapy for a targeted anxiety disorder caused positive benefits upon comorbid psychiatric symptoms [3, 10]. In order to provide more effective treatments, it is useful to examine baseline predictive factors of wide range of psychopathology after cognitive-behavioral therapy for panic disorder. Predictors of less effective treatment may save patients' time by avoiding ineffective treatment, which may be sometimes associated with economic burden. The previous study found that low levels of panic disorder symptom severity predicted positive treatment response for cognitive-behavioral therapy [11]. We found that some cognitive factors predicted several dimensions of psychopathology after cognitive-behavioral therapy for panic disorder [6]. However, research to identify predictive factors has been limited in cognitive-behavioral therapy for panic disorder, especially broad dimensions of psychiatric symptoms.

Patient personality characteristics are important in the treatment formulations of clinicians and researchers. Knowledge of a patient's personality trait may be useful in determining where psychological intervention should be provided, which type of group would be effective, and which psychological techniques should be emphasized. Some studies suggest that personality traits mediate broad dimensions of psychopathology. Ogrodniczuk et al. (2003) found that extraversion, conscientiousness, and openness were associated with favorable outcomes in group psychotherapy without cognitive-behavioral therapy [13]. Whether personality characteristics have an impact on cognitive-behavioral therapy outcomes is an important question. We found that openness, agreeableness, and conscientiousness at baseline predicted broad dimensions of psychopathology in social anxiety disorder patients after cognitive-behavioral therapy [14]. From the point of view of group therapy, our findings concerning openness and conscientiousness are consistent with those of Ogrodniczuk et al. [13]. In cognitive-behavioral therapy for panic disorder, however, few studies identified predictive personality traits [15, 16].

The present study aimed to examine the predictive value of personality traits for broad dimensions of psychopathology in panic disorder after cognitive-behavioral therapy.

## 2. Methods

### 2.1. Participants

Two hundred patients affected by panic disorder who attended the group cognitive-behavioral therapy program participated in the present study between October 2001 and May 2015. Some patients were referrals from the general medical or psychiatric clinics and departments and others who sought treatment for panic disorder themselves. All patients met the following inclusion criteria: (i) principal Axis I diagnosis of panic disorder according to the Diagnostic and Statistical Manual of Mental Disorders, Fourth Edition (DSM-IV) criteria, as assessed by the Structured Clinical Interview for DSM-IV(SCID) [17]; (ii) free from benzodiazepine use prior to cognitive-behavioral therapy entry, because these drugs may interact negatively with exposure treatments during cognitive-behavioral therapy [18, 19]; the established treatment manual that we used in this study contraindicated the benzodiazepine use in cognitive-behavioral therapy [20]; (iii) highly motivated to receive cognitive-behavioral therapy. We assessed the patient's motivation at interview. The patients had a variety of reasons for preferring this treatment. For example, insufficient effectiveness of drug therapy and refusal of medication owing to pregnancy. Exclusion criteria were current psychosis, bipolar disorder, and substance-use disorder. Use of antidepressants was allowed during the cognitive-behavioral therapy period since these drugs do not interfere with cognitive-behavioral therapy treatments [18]. Written informed consent was obtained from all patients after receiving a full explanation of the purpose and procedures of this study. The ethical approval was obtained from the ethics committee of our institute. The study was carried out in accordance with the Helsinki Declaration. The clinical study registration number in the Japanese trials registry is UMIN CTR 000031147.

### 2.2. Treatments

This cognitive-behavioral therapy program for the treatment of panic disorder was based on the established treatment manual developed by the Clinical Research Unit for Anxiety and Depression at the University of New South Wales, Sydney, Australia [20]. This treatment was conducted in groups of three to four participants by one principal therapist and one cotherapist. The therapist was a psychiatrist or a clinical psychologist with at least two years of clinical experience. The composition of the group was not influenced by panic disorder severity or other characteristics. The treatments comprised ten sessions, with each one lasting approximately 120 minutes. Each session was held once a week. The first two sessions included psychoeducation concerning the nature of anxiety, panic attack, panic disorder, and agoraphobia and provided a rationale for breathing retraining. From the third session, cognitive restructuring and situational and interoceptive exposure were introduced in session. The participants were assigned homework after each session.

### 2.3. Assessment

At baseline all the patients were assessed with the NEO Five Factor Index (NEO-FFI) to measure patient personality characteristics.

*NEO Five Factor Index (NEO-FFI). *The NEO-FFI is a 60-item self-reported instrument to measure the five personality dimensions of neuroticism, extraversion, conscientiousness, openness, and agreeableness [21]. There are 12 items per personality dimension. Each item is scored between 0 (strongly disagree) and 4 (strongly agree) [21]. Good reliability and validity of the Japanese version have been demonstrated [22].

All subjects were assessed with the following instruments at pre- and posttreatment.

*Symptom Checklist-90 Revised (SCL-90-R). *The SCL-90-R is a widely used assessment instrument for general psychopathology. It contains 90 items, subdivided into ten subscales of somatization, obsessive-compulsive, interpersonal sensitivity, depression, anxiety, hostility, phobic anxiety, paranoid ideation, psychoticism, and Global Severity Index. Each item is rated on a five-point scale ranging from 0 (not at all) to 4 (extremely) [23]. The average of the relevant items was taken to be the subscale score. The reliability and validity of the Japanese version have been reported [24].

*Agoraphobic Cognitions Questionnaire (ACQ). *The ACQ is a 14-item self-report tool to assess maladaptive thoughts concerning catastrophic consequences of experiencing anxiety. Each item is rated on a five-point scale ranging from 1 (thought never occurs) to 5 (thought always occurs), according to the occurrence with which this fearful thought occurred when the client became anxious. Adequate reliability and validity have been shown for the original and the Japanese versions of this measure [25, 26].

*Body Sensations Questionnaire (BSQ). *The BSQ is a 17-item self-report questionnaire to measure the fear evoked by panic-related bodily sensations. Each item is scored between 1 (not frightened or worried by this sensation) and 5 (extremely frightened by this sensation), according to the degree to which patients fear somatic symptoms. The reliability and validity of the original and the Japanese version of this questionnaire have been reported [25, 27].

*Panic Disorder Severity Scale (PDSS). *The PDSS is a seven-item scale for assessing overall severity of panic disorder in which the clinician rates the severity of seven features of panic disorder on a five-point scale ranging from 0 (none) to 4 (extreme). These seven areas contain the frequency of panic attacks, distress during the panic attacks, anticipatory anxiety, agoraphobic fear/avoidance, interoceptive fear/avoidance, work impairment/distress, and impairment of social functioning. The reliability and validity have been demonstrated for the original and the Japanese versions [28, 29].

### 2.4. Statistical Analyses

All statistical analyses were performed using SPSS 18.0 for Windows [30]. All the statistical tests were two-tailed and an alpha value of less than 0.05 was considered statistically significant. First, we used an independent samples *t*-test and *χ*^2^ tests to compare the demographic and clinical features between the participants who finished the program and those who did not. Second, we used paired *t*-tests to compare the baseline and endpoint symptom scores. Third, to examine the predictors of the indices of broad dimensions of psychiatric symptoms, we performed multiple linear regression analysis forcible loading method. We used the ten subscales of SCL-90-R at endpoint as dependent variables and age, sex, onset, and the total baseline scores of five personality dimensions of NEO-FFI, ACQ, BSQ, and PDSS as independent variables controlling the baseline subscales of SCL-90-R.

We conducted statistical analyses for these treatment outcomes based on the intention-to-treat principle with the baseline scores used as the last observations carried forward.

## 3. Results

### 3.1. Patients Characteristics

Table 1 summarizes the baseline demographic and clinical characteristics of participants. Twenty-eight patients (14.0%) out of the 200 who started the treatment dropped out before program completion. No statistically significant differences were seen between completers and dropouts.  Table 2 shows that all of SCL-90-R subscale scores at baseline were higher than those of Japanese community samples [12].

### 3.2. Pretreatment and Posttreatment Rating Scale Scores

Table 2 presents all the rating scale scores at pre-and posttreatment. All of the posttreatment scores were significantly lower than the pretreatment scores (*P* < 0.05).

### 3.3. Predictors of Broad Dimensions of Psychopathology

In multiple regression analysis (Table 3), NEO-FFI conscientiousness at baseline was a predictor of four SCL-90-R subscales at endpoint including obsessive-compulsive, depression, phobic anxiety, and Global Severity Index. ACQ score predicted anxiety; however BSQ score predicted nothing. Baseline PDSS total scores significantly predicted eight endpoint SCL-90-R subscales including obsessive-compulsive disorder, depression, anxiety, hostility, phobic anxiety, paranoid ideation, psychosis, and Global Severity Index.

## 4. Discussion

This study examined the relationship of personal traits at baseline and broad dimensions of psychopathology in patients with panic disorder after group cognitive-behavioral therapy using multiple regression analysis. The results showed that NEO-FFI conscientiousness score at baseline may predict some dimensions of psychiatric symptoms after treatment.

From the point of view of group therapy, our findings concerning conscientiousness are consistent with those of Ogrodniczuk et al. [13]. They found that conscientiousness was directly associated with favorable outcome in group psychotherapy. Our previous study found that conscientiousness predicted some dimensions of psychopathology in social anxiety disorder patients after cognitive-behavioral therapy [14]. Conscientiousness means a tendency to be well-organized, a trait of being well behaved, and a character by extreme care and great effort. In group therapy, patients with high conscientiousness probably work hard and endure discomfort [13]. Therefore the patients may have advantage in group psychotherapy including cognitive-behavioral therapy.

As mentioned above, patients affected by panic disorder commonly have broad psychiatric symptoms. From the clinical point of view, it may be useful to focus on conscientiousness at baseline for the purpose of improving broad dimensions of psychopathology in cognitive-behavioral therapy for panic disorder.

The dropout rate in this study was 14.0%. The previous meta-analysis showed that the average dropout rate was 19.6% in cognitive-behavioral therapy for anxiety disorder [31]. The rate in this study was lower than that in the previous review.

The present study has several limitations. First, the study did not involve a control group. We could not be sure if the significant reduction in broad dimensions of psychopathology may be due to group cognitive-behavioral therapy treatment for panic disorder rather than to passage of time. A randomized controlled trial with a control group is needed to examine the efficacy of the treatment. Second, we did not have follow-up data and were not able to decide the long-term effect on broad dimensions of psychopathology. Third, we used standardized cognitive-behavioral therapy manual; however, we did not record the interviews and performance to ensure the accuracy of materials provided to patients in the course of cognitive-behavioral therapy.

## 5. Conclusion and Recommendations

In summary, we examined the relationship of personal traits and broad dimensions of psychopathology in patients affected by panic disorder after cognitive-behavioral therapy using multiple regression analysis. Conscientiousness at baseline may predict several dimensions of psychiatric symptoms after treatment. For the purpose of improving broad psychiatric symptoms with patients affected by panic disorder, we need to pay more attention to conscientiousness trait at baseline.

## Figures and Tables

**Table 1:** Baseline characteristics of the patients.

Characteristics	Completers (*N* = 172)	Dropouts (*N* = 28)	*P* value
Female, number (%)	122 (70.9)	19 (67.9)	0.82
Mean age (SD)	36.1 (10.8)	33.8 (10.6)	0.30
Onset of panic disorder (SD)	29.1 (10.2)	28.3 (10.3)	0.68
Antidepressant use at baseline (%)	99 (57.6)	17 (60.7)	0.84
Current mood disorder (%)	9 (5.2)	3 (10.7)	0.38
Current anxiety disorder (%)	22 (12.8)	4 (14.3)	0.77
Current agoraphobia (%)	164 (95.3)	27 (96.4)	1.00
PDSS (SD)	13.2 (4.7)	12.4 (5.6)	0.44
ACQ (SD)	28.3 (9.4)	28.5 (10.4)	0.91
BSQ (SD)	46.3 (14.2)	42.8 (15.1)	0.24
NEO-FFI neuroticism (SD)	26.8 (9.1)	28.1 (6.9)	0.47
NEO-FFI extraversion (SD)	25.8 (8.2)	27.5 (7.0)	0.30
NEO-FFI openness (SD)	28.1 (6.2)	28.7 (6.2)	0.63
NEO-FFI agreeableness (SD)	32.6 (6.9)	32.2 (5.6)	0.76
NEO-FFI conscientiousness (SD)	27.3 (7.7)	28.0 (7.0)	0.68

*Abbreviations*. PDSS = Panic Disorder Severity Scale; ACQ = Agoraphobic Cognitions Questionnaire; BSQ = Body Sensations Questionnaire; NEO-FFI = NEO Five Factor Index.

**Table 2:** Pretreatment and posttreatment rating scale scores (*N* = 200).

	Community samples [12]	Pretreatment	Posttreatment	*P* value
PDSS (SD)		13.1 (4.8)	7.1 (4.7)	<0.05
ACQ (SD)		28.3 (9.5)	23.3 (7.9)	<0.05
BSQ (SD)		45.8 (14.3)	36.4 (13.7)	<0.05
SCL-90-R				
Somatization (SD)	0.63 (0.48)	1.18 (0.82)	0.84 (0.77)	<0.05
Obsessive-compulsive (SD)	0.72 (0.56)	1.14 (0.82)	0.85 (0.72)	<0.05
Interpersonal sensitivity (SD)	0.56 (0.52)	1.03 (0.79)	0.76 (0.68)	<0.05
Depression (SD)	0.73 (0.53)	1.15 (0.84)	0.82 (0.76)	<0.05
Anxiety (SD)	0.38 (0.39)	1.24 (0.87)	0.87 (0.80)	<0.05
Hostility (SD)	0.47 (0.50)	0.68 (0.68)	0.50 (0.57)	<0.05
Phobic anxiety (SD)	0.16 (0.30)	1.41 (0.92)	0.86 (0.85)	<0.05
Paranoid ideation (SD)	0.52 (0.52)	0.63 (0.69)	0.42 (0.51)	<0.05
Psychoticism (SD)	0.28 (0.38)	0.57 (0.57)	0.36 (0.44)	<0.05
Global Severity Index (SD)	-	1.04 (0.67)	0.73 (0.60)	<0.05

*Abbreviations*. PDSS = Panic Disorder Severity Scale; ACQ = Agoraphobic Cognitions Questionnaire; BSQ = Body Sensations Questionnaire; SCL-90-R = Symptom Checklist-90 Revised.

**Table 3:** Unique predictors of change in broad dimensions of psychopathology (*N* = 200).

	SOM	OC	INT	DEP	ANX	HOS	PHOB	PAR	PSY	GSI
Sex	−.02	−.01	.03	−.01	−.02	.06	−.01	.02	.01	−.00
Age	.13	.07	.01	.06	.13	.03	.16	−.06	.02	.09
Onset	−.07	−.01	.00	.00	−.11	−.05	−.17^*∗*^	.08	.03	−.05
PDSS	.04	.13^*∗*^	.11	.15^*∗*^	.13^*∗*^	.18^*∗∗*^	.14^*∗*^	.13^*∗*^	.14^*∗*^	.14^*∗*^
ACQ	.04	.04	−.04	−.02	.20^*∗*^	−.09	.01	−.04	.07	.05
BSQ	.07	.08	.05	.07	−.02	.12	−.04	−.01	−.05	.04
NEO-FFI										
Neuroticism	−.00	.05	.13	.10	.10	.05	−.00	.10	.07	.08
Extraversion	−.00	.02	.04	.06	.06	.09	−.04	.05	.09	.04
Openness	.02	.06	.04	.06	−.08	−.03	−.01	.04	.03	.02
Agreeableness	.03	−.01	−.01	.04	.05	−.06	.10	−.02	−.01	03
Conscientiousness	−.11	−.15^*∗∗*^	−.08	−.13^*∗*^	−.09	−.12	−.15^*∗*^	−.10	−.09	−.13^*∗*^
(Baseline score)	.63^*∗∗*^	.55^*∗∗*^	.55^*∗∗*^	.52^*∗∗*^	.45^*∗∗*^	.50^*∗∗*^	.64^*∗∗*^	.57^*∗∗*^	.62^*∗∗*^	.54^*∗∗*^

Adjusted *R*^2^	.49	.50	.46	.43	.42	.38	.49	.43	.51	.49

^*∗*^ *P* < 0.05;  ^*∗∗*^*P* < 0.01; the table shows the standardized beta coefficients. *Abbreviations*. PDSS = Panic Disorder Severity Scale; ACQ = Agoraphobic Cognitions Questionnaire; BSQ = Body Sensations Questionnaire; NEO-FFI = NEO Five Factor Index; SCL-90-R = Symptom Checklist-90 Revised; SOM = somatization; OC = obsessive-compulsive; INT = interpersonal sensitivity; DEP = depression; ANX = anxiety; HOS = hostility; PHOB = phobic anxiety; PAR = paranoid ideation; PSY = psychoticism; GSI = Global Severity Index.
